# Dental Pulp Reaction to Exposure at Different Time Intervals in Open Apex Canine Teeth of Cats

**Published:** 2009-04-17

**Authors:** Saeed Moradi, Maryam Bidar, Mohammad Hasan Zarrabi, Ali Talati

**Affiliations:** 1 *Department of Endodontics, Dental School, Dental Research Center, Mashad University of Medical Sciences, Mashad, Iran*; 2 *Department of Endodontics, Dental School, Dental Research Center, Mashad University of Medical Sciences, Mashad/ Iranian Center for Endodontic Research, Tehran, Iran*

**Keywords:** Cat, Dental Pulp, Inflammation, Open Apex

## Abstract

**INTRODUCTION:** Open-apex teeth with irreversible pulpitis require complex and difficult treatment. Providing the right environment for apexogenesis and pulp protection is vital for their long term prognosis. The young pulp of open apex tooth, however, is better equipped against irritation and assault. The aim of this study was to evaluate pulp inflammation in open apex canine teeth of cats.

**MATERIALS AND METHODS:** In this *in vivo* study, twelve cats with open apex canines were used. Pulps were iatrogenically exposed and the animals were sacrificed at one, seven, thirty and ninety days after pulp exposure. Samples were prepared for histological evaluations.

**RESULTS:** During the first and seventh day, changes were limited to acute inflammation in the coronal pulp. During the first month pulp changes in 45.5% of samples were similar to the seventh day. In the other samples necrosis and abscess spread to the end of the root, and internal resorption and periapical abscess were observed. In 45.5% of samples in the apical region vital tissue was barely observed (during 90 days); in 54.5% of samples however, complete pulp necrosis, internal resorption and a large periapical lesion was observed.

**CONCLUSION:** In one and seven-day periods pulp tissue was vital, however, in the thirty and ninety-day periods, minority of the pulp samples were vital.

## INTRODUCTION

Dental pulp is a mesenchymal-originated soft tissue that occupies the pulp chamber and root canal space and contains specialized cells on its outer surface called ‘odontoblasts’ (1).

During vasodilatation, increase capacity and blood vessel permeability of pulp is limited because of the dentin that completely surrounds the dental pulp. At this time, proper regulation of blood flow is very important and critical (1).

Bacterial, chemical and mechanical irritations and injury can affect the dental pulp. Microorganisms are one of the etiological factors of dental pulp diseases. Kakehashi *et al.* created dental pulp exposure in normal and germ-free rats. In normal (not in sterile) rats pulpal and periradicular lesions were observed (2). It has been shown that even a small injury in enamel can affect the pulp health (3). Enamel and dentin caries contain different bacteria like Lactobacillus*, *Actinomycosis* and *Streptococcus (4).

Direct and indirect trauma also affect the dental pulp, therefore endodontic follow up is important (5). Most dental traumas to permanent teeth occur during 7-15 years (6); trauma can affect the growth and development of these teeth (7).

In large coronal caries with pulp exposure, the adjacent pulp tissue is inflamed and liquefaction necrosis with an infiltration of PMNS occurs (8). This inflammation may be sustained for long periods with pulp necrosis occurring gradually; on the other hand the inflammation may cause necrosis rapidly.

Various factors affect this process *e.g.* the microbial virulence, host resistance, blood supply, and lymphatic drainage. A study performed on pulpal exposure of rats demonstrated that the spread of necrosis within the pulp occurs in a coronal-apical direction, in the periapical lesion it is directed in horizontal and then vertical route (9).

**Figure 1:**
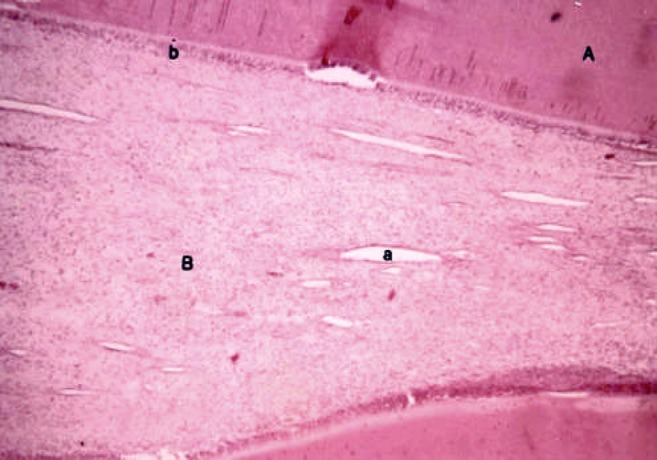
One day sample; A: Dentin, B: Pulp, a: Dilated arteriole, b: Odontoblastic layer

Pulpal damages may finally cause cell death, releasing a whole host of chemical mediators (10). In addition to a nonspecific inflammatory reaction, immunologic reactions also cause continuation of inflammation (11-12). Consequently, permeability and blood vessel stasis increase. If the removal of liquids from the lymphatic system is not adequate, complete venol collapse may occur (13-14).

Open-apex teeth with irreversible pulpitis require complex and difficult treatment. Protecting the dental pulp via providing suitable conditions for its growth can affect the prognosis of such teeth. The type and duration of injury are also noteworthy factors in the prognosis and treatment planning process (5). The time interval between injury and clinical examinations may affect the health of dental pulp (15). It is generally believed that open apex teeth have greater resistance than mature teeth providing more effective protection against irritations; this may be due to their ability to regenerate or repair damaged pulpal tissue (16). Open-apex teeth showed revascularization after 2 hours; this however, is not feasible in mature teeth (17). Numerous studies investigated pulp and periapical inflammation of close-apex teeth (8,9,15,18-21), while such information are not assessed and clarified well about teeth with open apices; therefore, the aim of this *in vivo* study was to evaluate pulpal inflammation involving open- apex canine teeth of cats.

**Figure 2:**
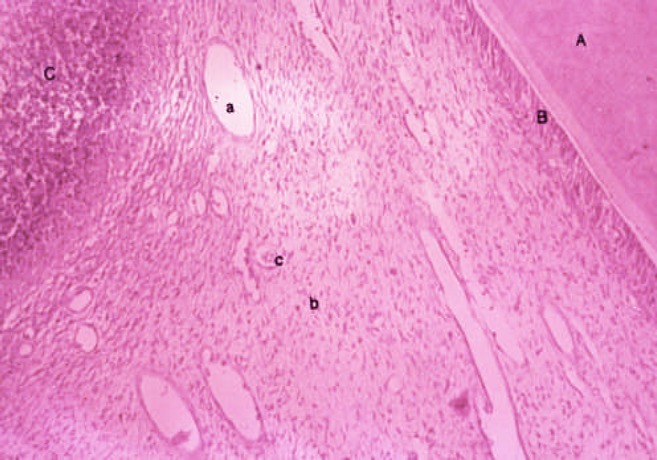
Seven days sample; A: Dentin, B: Odontoblastic layer, C: Inflammatory cells in exposure zone, a: Dilated arteriole, b: Fibroblasts, c: PMN cell

## MATERIALS AND METHODS

This study is confirmed by ethical committee of Mashad University of Medical Sciences. Twelve cats with no contributory systemic disease were selected for this study. The four open-apex canine teeth of each cat were included. Exclusion criteria included closed apex and traumatized teeth. Animals were rendered unconscious with a muscular injection of Ketamin HCl (10mg/kg) and Xylazin (1mg/kg). After taking radiography and confirming the opening of the apices of canine teeth, 1 mL 2% Lidocaine with 1:100000 epinephrine was used for local anesthesia. Pulpal exposure was made using 0.5-mm round bur with a high speed handpiece, one tooth left intact as a control. Vital perfusion was performed after 1, 7, 30 and 90 days. Each period had 11 teeth for the test and one tooth for control. At each time, 11 teeth were considered as experimental and one tooth as a control sample.

Block sections were made from canines containing their periodontium and a part of surrounding healthy bone. Samples were immersed in 10% neutral buffered formalin and were then decalcified and embedded in paraffin. All samples were stained with hematoxylin and eosin for the purpose of histological evaluations.

## RESULTS

Histological evaluations did not show any inflammation in pulp and periapical tissues of control teeth.

**Figure 3:**
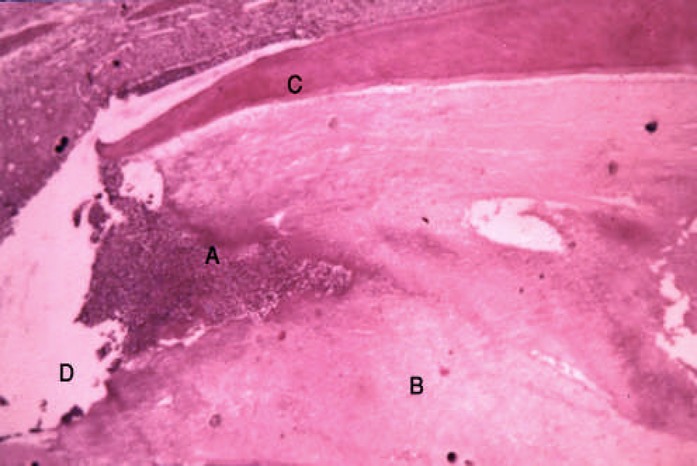
Thirty days sample; A: Inflammatory cells in exposure zone, B: Pulp, C: Dentin, D: Exposure zone

The results of the 1^st^ day showed changes in all samples at the exposed parts of the pulps, while other parts were intact. These findings showed acute inflammation near the exposed region with absence of any granulation tissue. Mild hyperemia was detected in remaining pulp and odontoblastic layer remained intact. No internal or external resorption was detected (Figure 1).

Seven-day specimens showed acute and chronic inflammation in addition to abscess beneath granulation tissue. Inflammation was limited to coronal pulp. The exposed region demonstrated necrosis; majority of blood-vessels were dilated and the odontoblastic layer was destructed. Internal resorption was not observed (Figure 2).

In 45.5% of the 30^th^ day sample observations were similar to the 7^th^ day. Extension of abscess and granulation tissue to the apical regions were observed. No internal resorption was noted or periapical lesion (Figure 3). In another 45.5%, the necroses and abscess spread to the end of the root; internal resorption and periapical granulation tissue were also observed. In remaining 9% of samples, pulps were completely necrosed with the formation of apical granulation tissue. There was no internal resorption, also acute and chronic inflammatory cells were observed.

**Figure 4:**
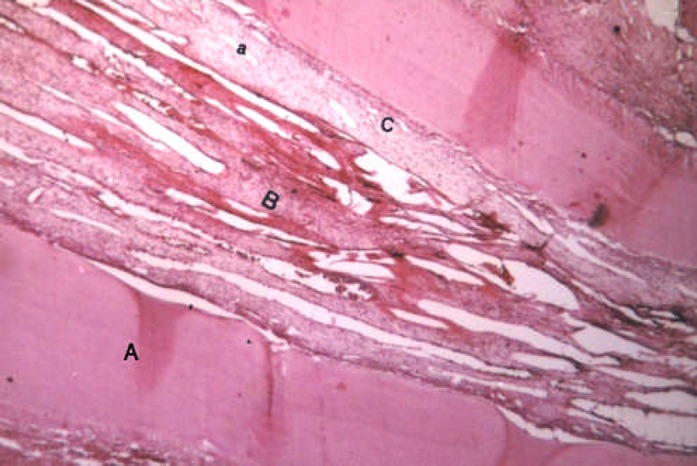
Ninthy days sample; A: Dentin, B: Degenerative Pulp, C: Pulp is about to generation, a: Dilated arteriole

At the 90^th^ day 54.5% of samples demonstrated total necrosis of dental pulps i.e. microorganisms and debris were found with further internal resorption, and extended periapical lesion (Figure 4). In the remaining 45.5% of samples, minute amounts of vital tissue were observed in apical region of canals. Severe infiltration of acute and chronic inflammatory cells was also observed in granulation tissue.

## DISCUSSION

The aim of this *in vivo* study was to determine the relation of inflammation with post-exposure time lapse in open-apex teeth. Also the type and extent of inflammation at different post-exposure time intervals was compared.

Cats were chosen because of morphologic similarity to human canines and their relatively simple maintenance similar to Torabinejad’s study (22). However, for selecting animals, researchers must consider that animals should be of a mammalian family that is not too dissimilar to humans in evolutionary terms. Secondly, though all results of animal studies cannot be generalized to humans, this information can be practical and helpful for conducting successful treatments. Despite the similarities of cat studies with human studies, some problems do exist *e.g*. selecting a cat with suitable age and open-apex tooth, probability of cat's death because of inflammation/infection, swelling of jaw during the process of unconsciousness. This study showed that after 1 and 7 days, vital hyperemia was only observed in deeper parts of the pulp tissue. Conservative treatments in such cases can be more efficient.

The results of this study at 7^th^ day concur with a similar study carried out by Torneck *et al.* (23).

A limitation in this study may the long period that teeth were not assessed between one week and one month intervals. Therefore, samples should be observed between 1^st^ week and the 1^st^ month to achieve a more precise evaluation of pulpal changes. In a study by Yamasaki *et al.* the inflammation of the coronal pulp extended towards the apex on the 14^th^ and 21^st^ day (9).

## CONCLUSION

Even when considering the limitations of this *in vivo *study using the open-apex canine teeth of cats as an apexogenesis model, the first seven days are the most essential for maintaining tooth vitality after pulp exposure.
